# Current status of neural progenitor/stem cells for spinal cord injury: fundamental research and clinical trials

**DOI:** 10.3389/fneur.2026.1772547

**Published:** 2026-05-25

**Authors:** Xin-xin Chen, Zhe Yin, Xiu-hang Zhang, Jie Hao, Qi Zhou, Jia-ao Yu, Jun Wu

**Affiliations:** 1 Department of Burns, The First Hospital of Jilin University, Changchun, China; 2 State Key Laboratory of Organ Regeneration and Reconstruction, Institute of Zoology, Chinese Academy of Sciences, Beijing, China; 3 National Stem Cell Resource Center, Institute of Zoology, Chinese Academy of Sciences, Beijing, China; 4 Institute for Stem Cell and Regenerative Medicine, Chinese Academy of Sciences, Beijing, China; 5 Beijing Institute for Stem Cell and Regenerative Medicine, Beijing, China; 6 University of Chinese Academy of Sciences, Beijing, China

**Keywords:** cell therapy, clinical trial, neural progenitor cells, spinal cord injury, transplantation

## Abstract

**Background:**

Spinal cord injury (SCI) causes irreversible neurological deficits and represents a major global health and socioeconomic burden. Although neural progenitor cell (NPC) transplantation is strongly supported by preclinical evidence through cell replacement, intrinsic neuroregeneration, and broad neurotrophic and immunomodulatory effects, its clinical translation has progressed more slowly than anticipated. In parallel, rapid advances in gene editing, biomaterial engineering, and organoid technologies are reshaping the therapeutic landscape. Therefore, it is timely to systematically re-evaluate the current evidence on NPC-based therapies for SCI and to refine future translational strategies.

**Main body:**

This review provides an updated and comprehensive overview of NPC therapy for SCI across the full translational continuum. First, we summarize the biological properties, advantages, and limitations of NPCs derived from adult or embryonic neural tissues, embryonic stem cells (ESCs), and induced pluripotent stem cells (iPSCs), highlighting issues such as tumorigenicity, immune responses, and manufacturing standardization. We then focus on efficacy-oriented genetic modifications and engineered delivery systems, including NPCs overexpressing neurotrophic, synaptogenic, or pro-survival factors, as well as combination strategies integrating NPCs with biomaterials, small molecules, or immunomodulatory agents to enhance graft survival, circuit reconstruction, and motor and sensory recovery. Subsequently, we systematically analyze 19 clinical trials of NPC/NSC-based products conducted in 10 countries, covering HuCNS-SC, LCTOPC1, NSI-566, hESC-OPC, and the emerging iPSC-derived product XS228. Trial designs, dosing regimens, routes of administration, safety profiles, and preliminary functional outcomes are compared, and key design principles for next-generation clinical trials and patient selection are proposed. Finally, we discuss organoid-based approaches and artificial intelligence (AI)–assisted decision tools as emerging platforms for disease modeling, protocol optimization, and precision indication refinement.

**Conclusion:**

NPC-based therapy for SCI remains at an early but promising translational stage. No NPC product has yet achieved regulatory approval, reflecting persistent challenges in cell source optimization, safety control, graft survival, *in vivo* tracking, and trial design. Nevertheless, by integrating rigorous cell source selection, rational gene modification, advanced delivery systems, and well-designed clinical trials targeting carefully defined patient populations, NPCs are expected to achieve meaningful—and potentially transformative—clinical benefits for individuals with SCI in the future.

## 1 Introduction

Spinal cord injury (SCI) is a common and devastating condition that severely affects patients' physical and psychological well-being. Due to the need for ongoing medical care and rehabilitation, SCI imposes a substantial healthcare burden worldwide. Epidemiological data on SCI incidence are limited and vary widely across regions: for example, the highest reported incidence in Canada is 1,298 per million population, whereas Finland reports only 280 per million (1, 2). According to the World Health Organization, an estimated 15.4 million individuals worldwide were living with SCI in 2021. To date, significant progress has been made in understanding the pathological evolution of SCI.

The pathophysiology of SCI comprises primary and secondary injury mechanisms. Primary injury leads to hemorrhage within the spinal gray matter, causing local ischemia and hypoxia and subsequent neuronal loss and demyelination. Additionally, progressive edema may further impair neural conduction (3). Compared with primary injury, secondary injury represents a progressive cascade of pathological events, including neurogenic shock, vascular disruption, excitotoxicity, calcium-mediated damage, electrolyte imbalance, immune-mediated injury, apoptosis, and mitochondrial dysfunction (4).

These combined mechanisms ultimately result in irreversible spinal cord damage and permanent neurological deficits (5). Temporally, irreversible damage occurs in the gray matter within 1 h after injury, whereas the white matter becomes irreversibly impaired within 72 h (6). Due to progressive tissue destruction and the minimal intrinsic regenerative capacity of the adult spinal cord, spontaneous neurological recovery after SCI is extremely limited. To date, there is still no cure for SCI. Thus, regenerative strategies aimed at restoring neurons and glial cell populations represent a highly promising therapeutic avenue (3, 7, 8).

Neural progenitor cells (NPCs) are multipotent precursor cells within the central nervous system that give rise to diverse neuronal and glial cell types. NPCs encompass both neural stem cells and downstream progenitor subpopulations (9, 10). Neural stem cells have unlimited self-renewal capacity and generate NPCs, which possess restricted proliferative potential and can differentiate into neurons, astrocytes, and oligodendrocytes (11). Beyond lineage differentiation, NPCs can migrate, integrate into host tissue, establish gap junctions, regulate the microenvironment, and contribute to the restoration of tissue homeostasis. Moreover, NPCs secrete glial cell-derived neurotrophic factor (GDNF), BDNF, CNTF, IGF-1, and other trophic factors that enhance cell survival and suppress neuroinflammation (12–16).

With strong capacities for proliferation and differentiation, low tumorigenicity, ease of integration into host tissue, minimal immune rejection, and amenability to genetic modification, NPC-based therapy holds tremendous potential for repairing damaged spinal cord tissue. Preclinical studies have demonstrated both the safety and functional benefits of NPC transplantation (17–19).

Despite the strong rationale supporting NPC-based therapy—through cellular replacement, intrinsic neuroregenerative mechanisms, and broad neurotrophic effects—clinical translation remains slower than anticipated. Therefore, there is an urgent need to systematically evaluate existing evidence, identify current challenges, and explore innovative strategies such as gene-modified cell therapy and advanced biomaterial-based delivery systems to optimize therapeutic effectiveness.

This review provides a comprehensive overview of the current progress in NPC-based therapy for SCI, from preclinical development to clinical applications. It highlights gene-modified NPC technologies and engineered delivery systems that are closely linked to therapeutic outcomes. In addition, emerging approaches—including organoid technology and artificial intelligence (AI)—are discussed as future directions to enhance the feasibility and efficacy of NPC-based interventions for SCI.

## 2 Sources of NPCs for spinal cord injury therapy

Neural progenitor cells used for spinal cord injury (SCI) therapy can be categorized into three major sources: adult or embryonic neural tissues, embryonic stem cells (ESCs), and induced pluripotent stem cells (iPSCs) (20).

Adult or Embryonic Neural Tissue-Derived NPCs: to meet the demand for large-scale *in vitro* expansion, adult neural progenitor cells (aNPCs) are typically harvested from the subventricular zone of the brain or spinal cord. Under culture conditions supplemented with epidermal growth factor (EGF) and/or basic fibroblast growth factor (bFGF), these cells can be enriched and expanded as neurosphere NPC aggregates. After several passages, they are capable of differentiating into neurons, astrocytes, and oligodendrocytes (21).

ESC-Derived NPCs: ESC-derived NPCs originate from the pluripotent inner cell mass of embryos and possess the ability to form derivatives of the ectoderm, mesoderm, and endoderm. Although several research groups have developed protocols to differentiate ESCs into transplantable neural or glial cells, the efficiency of conventional methods remains limited. The addition of Noggin protein and the small-molecule inhibitor SB431542 has been shown to enhance NPC induction efficiency and shorten the neural differentiation timeline—reducing the induction period from 30–50 days to approximately 19 days (22, 23).

iPSC-Derived NPCs: iPSCs are generated through the reprogramming of somatic cells back to a pluripotent state, functionally resembling ESCs (24). This approach not only avoids the ethical concerns associated with using embryonic or adult neural tissues but also enables the development of patient-specific autologous cell therapies, which may reduce or eliminate the lifelong need for immunosuppression following transplantation (25). Due to their accessibility, autologous transplant compatibility, and strong neuroregenerative potential, iPSC-derived NPCs are considered one of the most promising cell sources for SCI treatment. However, the technology still faces challenges. Immune rejection may occur even when cells are autologous, as reprogramming and differentiation processes can introduce neoantigens or residual vector components that trigger immune responses. Tumorigenicity and potential genomic instability remain major safety concerns, requiring further clinical verification.

Recent clinical trials using iPSC-derived NPCs have provided more robust evidence supporting their therapeutic potential (26). Mechanistic studies indicate that grafted cells promote repair predominantly through two pathways: differentiation into functional neural cells to replace damaged tissue, and modulation of the local inflammatory microenvironment. Future therapeutic strategies are expected to balance personalization and standardization, enhancing safety and reproducibility by optimizing cell manufacturing processes and surgical delivery protocols (Tables 1, 2).

**Table 1:** The effect of different sources of NP/SCs on spinal cord injuries.

Cell type	Ethical issue	Safety risk	Tumorigenicity	Isolation	Pre-isolation storage	Post-isolation storage	Transfection	Therapeutic effects	References
NS/PCs 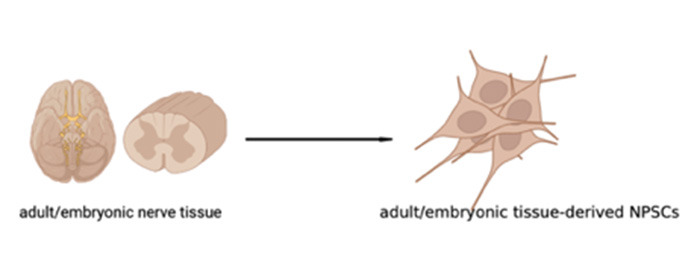	+	+	–	+	–	+	+	To improve the NF secretion	Kobayashi et al. (119)-a. improve the NF secretion c. inhibit demyelination Kawabata et al. (120)-b. enhance axonal regrowth c. inhibit demyelination d. promote synapse formation Okubo et al. (121)-b. enhance axonal regrowth d. promote synapse formation Salewski et al. (122)-f remyelination Li et al. (123)-g. improve respiratory function Ito (57) a. improve the NF secretion b. enhance axonal regrowth h. enhance motor functional recovery Ruzicka et al. (124)-e inhibit glial scar Kawabata et al. (120)-f. remyelination
								To enhance axonal regrowth	
								To inhibit demyelination	
								To promote synapse formation	
								To inhibit glial scar	
								To remyelination	
								To improve respiratory function	
								To enhance motor functional recovery	
								To reduce lesion size	
ESC-NS/PCs 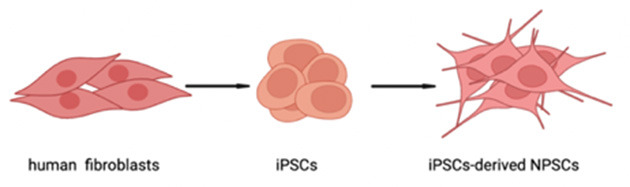	–	+	+	+	–	+	+	To improve cell proliferation	Yamane et al. (125)-j improve cell proliferation
								To increase myelination	Iwai et al. (126)-k. increase myelination n. form synaptic connections
								To modulate the inflammatory response	Lu et al. (51)—anti-inflammatory modulation
								To increase neuroprotective cytokines	Morizane et al. (86)—safe iPSC-NS/PCs
								To form synaptic connections	Jain et al. (87)—synaptic reconnection
iPSC-NS/PCs 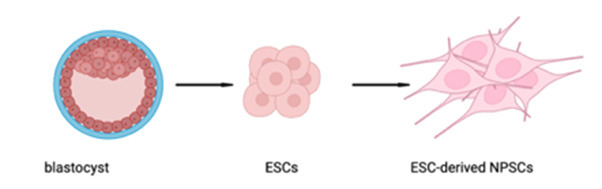	–	?	+	+	–	+	+	To relief neuropathic pain	Morizane et al. (86) neuropathic pain relief
								To empower neuros	Levi et al. (66) neuronal regeneration
								To promote astrogliosis	Ben-David and Benvenisty (88) astrocytic bridging

**Table 2:** Comparison of major sources of neural progenitor cells for spinal cord injury therapy.

NPC source	Differentiation efficiency	Tumorigenicity risk	Immune rejection intensity	Production cost	Standardization difficulty
Adult/embryonic neural tissue	High (>70%, but with significant batch-to-batch variability) (89, 90)	Very low (89, 91)	Moderate (91–93)	High (limited tissue availability, low yield) (91–93)	High (large batch variability, difficult to standardize) (91–93)
ESCs	High (60–90%) (89, 90, 94)	Moderate (risk of residual undifferentiated cells; requires strict purification) (88, 95–97)	High (allogeneic transplantation requires immunosuppression) (92, 96, 98)	Moderate (well-established culture systems, but ethical constraints) (92, 96)	Moderate (variability among ESC lines; requires standardized protocols) (93, 96)
iPSCs	Moderate to high (40–80%, with significant line-to-line variability) (87, 89, 94, 99)	Moderate (tumorigenic potential in certain subpopulations; requires purification and validation) (88, 100, 101)	Low (lowest in autologous transplantation; allogeneic use requires HLA matching or engineering) (86, 92, 102)	Low (amenable to large-scale production, though processes are complex; costs decreasing) (87)	Moderate (line variability and reprogramming methods affect standardization) (87, 94)

## 3 Historical development of NPC-based therapy for spinal cord injury

**Figure 1:**
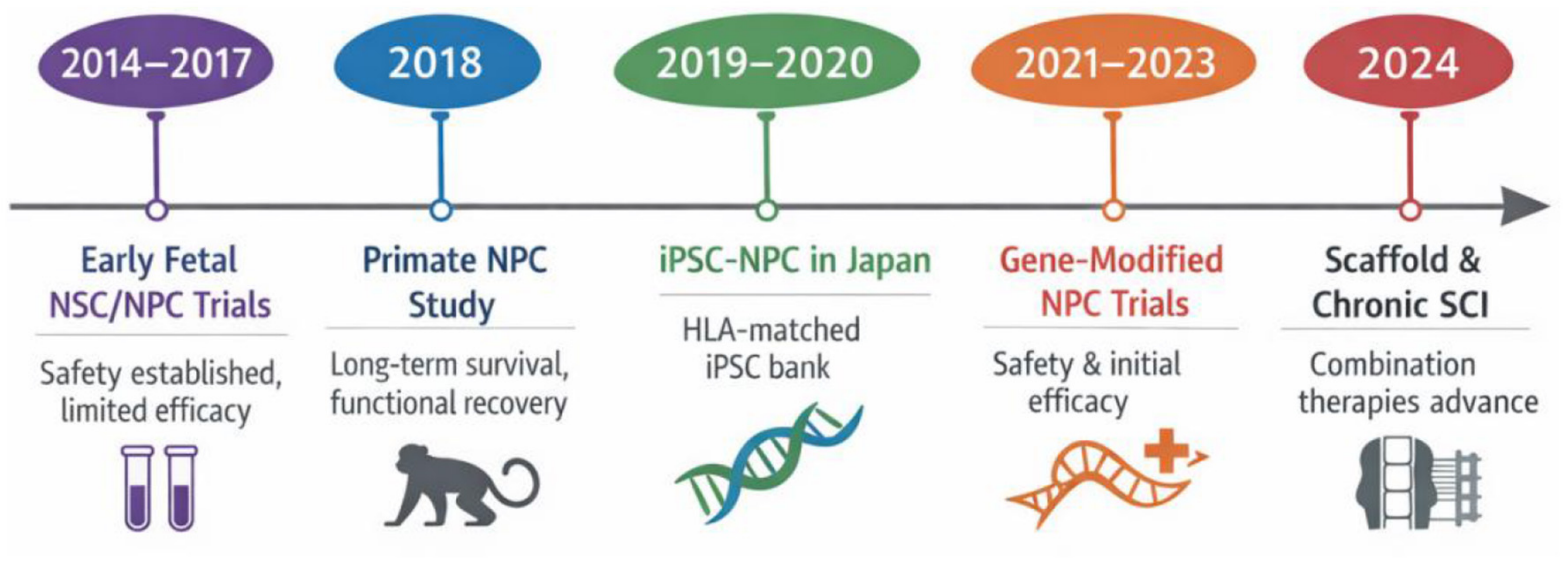
Timeline of key translational advances in NPC/NSC-based therapy for spinal cord injury.

**Figure 2:**
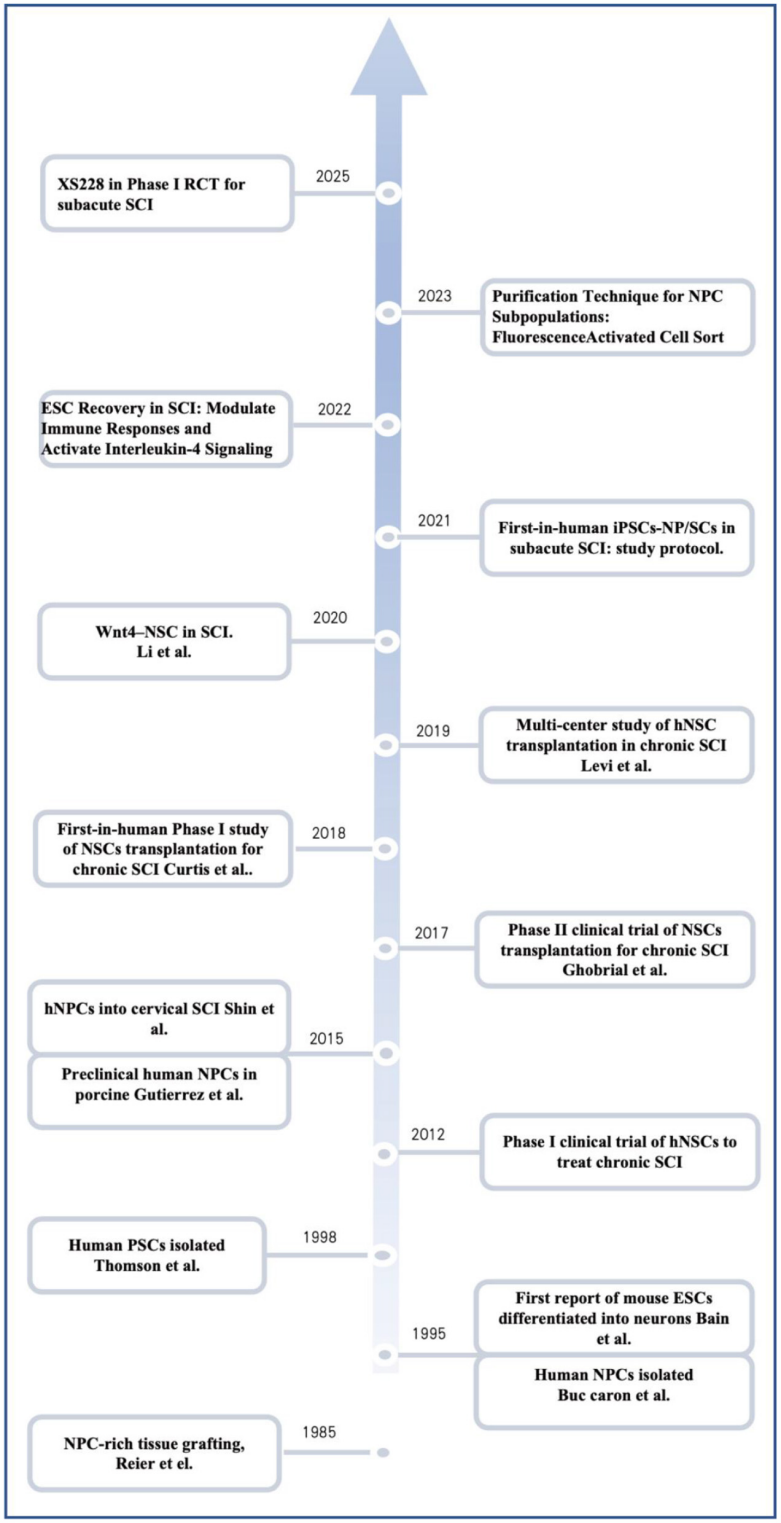
The timeline of major advances during the spinal cord injury therapy via NP/SCs. 1980s—NPC-rich tissue grafting (103); 1995—first report of mouse ESCs differentiated into neurons for transplantation (104); 1995—Human NPCs isolated (105);1998—human PSCs isolated (106, 107); 2012—phase I clinical trial of hNSCs to treat chronic SCI (29). 2015—preclinical human NPCs in porcine (27). 2015—hNPCs into cervical SCI (68). 2017—Phase II clinical trial of NSCs transplantation for chronic SCI (30). 2018—first-in-human phase I study of NSCs transplantation for chronic SCI (66). 2019—multi-center study of hNSC transplantation in chronic SCI. 2020—Wnt-NSC (28). 2021—first in human iPSC-derived NS/PCs in subacute complete spinal cord injury: study protocol (38). 2022—ESC recovery in SCI: modulate immune responses and activate interleukin-4 signaling (63). 2023—purification technique for NPC subpopulations: fluorescence activated cell sort (77). 2025—XS228 in Phase I RCT for subacute SCI.

In the early stages of SCI treatment, Reier first proposed the use of neural tissues rich in NPCs and achieved favorable therapeutic outcomes. This represented a pivotal milestone in the evolution of purified cell-based treatment strategies (27). In 1995, researchers successfully isolated and expanded murine NPCs *in vitro* for transplantation, followed by the successful differentiation and application of human NPCs, which also demonstrated promising functional improvements in SCI models. Gutierrez further evaluated the safety of direct intramedullary cell transplantation in a porcine SCI model, investigating the tolerance of the spinal cord to increased injection volumes and multiple delivery sites. In 2010, Geron launched the first clinical trial of NPC transplantation for SCI using human ESC-derived oligodendrocyte progenitor cells (OPCs) injected directly into injured spinal lesions. This trial paved the way for translating NPC-based therapy into human applications. Early clinical studies primarily focused on chronic SCI. Recent advancements have enabled fluorescence-activated cell sorting (FACS) to purify specific NPC subpopulations with greater precision (20). As of March 2025, K Pharma in Japan has received approval to initiate a larger-scale clinical trial, and SciSET Bio in China is progressing into a registered Phase I clinical study—advances expected to provide more robust and reliable efficacy data. To date, more than a dozen clinical trials have been identified, covering acute and subacute SCI, thoracic and lumbar injury sites, and multiple NPC sources (28–30). Key parameters—including transplantation site and timing, cell dose, graft survival, migration, differentiation, and functional recovery—are comprehensively discussed in the clinical trial section of this review (31–34). In recent years, translational research on NPC/NSC-based therapy for SCI has accelerated further, gradually shifting from early safety validation toward more engineered and precision-oriented interventions. Between 2014 and 2017, early phase I/II clinical trials, mainly based on fetal-derived NPCs, were successively conducted, providing preliminary evidence of feasibility and safety, although overall efficacy remained limited (35, 36). In 2018, studies in non-human primate models further demonstrated that transplanted human NPCs could achieve long-term survival and promote forelimb functional recovery, thereby providing important preclinical support for subsequent clinical translation (37). During 2019–2020, iPSC-derived NPCs were first approved for clinical testing in Japan, while the establishment of HLA-matched iPSC banks offered a new strategy to reduce immune rejection in allogeneic transplantation (38, 39). From 2021 to 2023, multiple studies of iPSC-NPC transplantation in patients with subacute SCI were initiated, with a major focus on safety and preliminary efficacy evaluation; meanwhile, gene-modified NPCs, such as GDNF-engineered cells, showed enhanced effects on neural differentiation and functional recovery (40). By 2024, the combination of biomaterial scaffolds with NPC/NSC transplantation had emerged as an important direction in the field, and several systematic reviews and network meta-analyses suggested that this strategy may be superior to cell therapy alone (41). In parallel, new progress was also achieved in interventions for chronic SCI and rehabilitation-based combination strategies. Collectively, these advances indicate that NPC/NSC therapy is evolving from simple cell transplantation toward a new stage characterized by cell engineering, biomaterial integration, and comprehensive therapeutic strategies (42, 43) (Figures 1, 2).

## 4 Genetically modified NPCs for SCI therapy

### 4.1 NPCs overexpressing neurotrophic factors

Genetically modified neural progenitor cells (NPCs) that overexpress neurotrophic factors can promote neuronal differentiation, enhance axonal regeneration, and improve the lesion microenvironment, thereby significantly facilitating functional recovery after spinal cord injury (SCI) (40, 44).

- GDNF

GDNF is a representative neurotrophic factor used to enhance NPC-based therapy for SCI. In a rat thoracic contusive SCI model, adenovirus-mediated GDNF overexpressing human fetal brain-derived hNSPCs were transplanted intramedullarily at 7 days after injury (total volume: 12 μl; cell concentration: 8.0 × 10^4^ cells/μl). The results showed that GDNF overexpression enhanced neuronal differentiation *in vitro* (Tuj1: 39.4% ± 3.7% vs. 25.4% ± 3.0%) and improved *in vivo* repair, as evidenced by reduced lesion volume (1.6 ± 0.3 mm^3^ vs. 4.3 ± 0.2 mm^3^ in the vehicle group), decreased demyelinated volume (14.0% ± 1.4% vs. 29.4% ± 1.5%), and higher BBB scores at 9 weeks after transplantation than those in the control-transduced hNSPC group (7.8 ± 0.6 vs. 5.8 ± 0.5) (45–47).

- NEP1-40

In an *in vivo* rat T9 hemisection SCI model, NEP1-40 gene-modified NSCs were transplanted directly into the lesion site at 7 days after injury. Compared with unmodified NSCs, NEP1-40-modified NSCs significantly increased the number of HRP-positive nerve fibers at 8 weeks after transplantation (59.25 ± 7.75 vs. 33.58 ± 5.47), indicating enhanced axonal regeneration. They also improved the expression of neuronal and myelin-related markers, including NF-200 and MBP. However, NEP1-40 did not directly alter lineage-specific differentiation, as the proportions of NF-200-, MBP-, and GFAP-positive cells were not significantly different from those in the unmodified NSC group (48).

- NT-3

**Figure 3:**
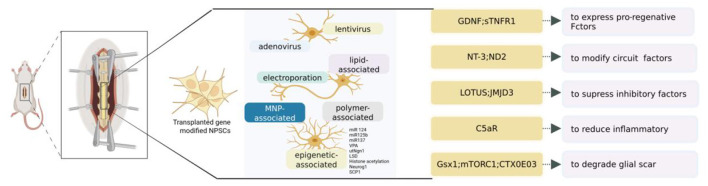
Application of genetic modified NP/SCs as promising cells for the SCI treatment in preclinical trials. Notch1 (108, 109) Wnt (110).

Recent studies suggest that overexpression of growth factors can enhance axonal regeneration. Neurotrophin-3 (NT-3), a member of the neurotrophin family, has shown great promise for SCI treatment. Lu et al. demonstrated that NT-3 modification promoted survival/proliferation, neuronal differentiation, and neurite outgrowth of hfNSCs *in vitro* through high-affinity binding to tropomyosin receptor kinase C (TrkC). Eight weeks post-infection, 37.9 ± 4.2% of hfNSCs in the Retro-NT-3 group expressed neuronal markers, significantly higher than those in the control and mock groups (49–52) (Figure 3 and Table 3).

**Table 3:** hNP/SCs-based modified cell in treatment of SCI.

Gene-modified NP/SCs	Preferred receptor	Gene editing strategies	Therapeutic effect	PMID	Author
microRNA-125b-Rat-NSC	Smurf1/KLF2/ATF2	Lentivirus mediated	Motor function recovery	33951295	Zhao et al. (111)
			BBB (treatedgroup:0W:4; 1W:8; 2W:9; 3W:12; 4W:15 controlgroup: 0W:2; 1W:4; 2W:5; 3W:6; 4W:7)		
JMJD3-NPC	SAPK/JNK	GKS-J4 treated	Promotes oligodendrocyte-lineage commitment post SCI	33428096	Zhang et al. (58).
TrkC-Rat-NSC	NT-3	Adenoviral (Ad) vectors expressing GFP-TrkC	Motor function recovery	3389887	Li et al. (52)
			BBB (treatedgroup: 0W:0; 1W:1; 2W:2; 3W:3; 4W:4.5; 5W:5; 6W:5.5; 7W:6.5; 8W:7 controlgroup: 0W:0; 1W:0; 2W:0.5; 3W:1; 4W:1.5; 5W:1.5; 6W:2; 7W:3; 8W:3)		
hGDNF/sTNFR-NSC I	GDNF/sTNF-αI	Adenoviral (Ad) vectors mediated	Neural stem cell differentiation	22840455	Zhuang et al. (112)
Gsx1-	Notch; Nanog; Wnt	Lentivirus-transduced	Cell survival and proliferation	33895323	Patel et al. (113)
			Motor function recovery		
			BMS (treated group:0W:0; 1W:3; 2W:4; 3W:4.5; 4W:5; 5W:6.5; 6W:7; Control group: 0W:0; 1W:1; 2W:1.5; 3W:1.5; 4W:2; 5W:2.5; 6W:3)		
Wnt4—NSC	β-catenin/MAPK/JNK	Lentivirus carrying GFP and Wnt4 gene	Repairing injured spinal cord; restoring hindlimb motor function	31914702	Li (59).
LOTUS—hiPSC-NS/PCs	RhoA/ROCK	Lentivirus mediated	Increase the secretion of neurotrophic factors, enhance motor functional recovery	34653401	Ito (57)

### 4.2 NPCs overexpressing synaptogenic factors

NPCs engineered to overexpress synaptogenic molecules can promote synaptic formation, strengthen synaptic connections between grafted cells and host neurons, and facilitate neural network reconstruction, thus improving functional outcomes after SCI (53, 54).

- ND2

Genetic modification of NPCs using bHLH transcription factors such as NeuroD2 (ND2) enhances their differentiation into disease-relevant neuronal subtypes. Overexpression of ND2, Neurogenin1, and Neurogenin2 in N18-RE-105 neuroblastoma cells induces neuronal morphological differentiation and increases synaptic protein expression. Notably, ND2 promotes neurite formation and upregulates synaptic proteins such as synaptotagmin—normally not expressed in this cell type—and redistributes SNAP25 to the plasma membrane. In addition, the neuron-specific isoform of tubulin and synaptic target proteins are significantly increased in ND2-overexpressing cells (55).

- mTORC1

In a stroke model induced by middle cerebral artery occlusion (MCAO), transplantation of CTX0E03 cells resulted in significant improvements in sensorimotor function and reduced motor asymmetry at 6–12 weeks post-transplantation. These findings suggest a critical role of mTORC1 signaling in NPC activation (31). Studies in Xenopus further revealed that mTORC1 may contribute to spinal cord regeneration by supporting intrinsic axonal regrowth mechanisms. This may explain the reduced number of regenerating axons after mTOR inhibition. Impaired functional swimming recovery may thus be attributed to disrupted mTORC1 signaling, highlighting this pathway as a potential target for enhancing axonal regeneration in future studies (56).

- LOTUS

One study evaluated *ex vivo* gene therapy using lentiviral LOTUS-overexpressing hiPSC-derived NS/PCs for subacute SCI. After *in vitro* differentiation, LOTUS-NS/PCs exhibited longer neurites than control cells and reversed NgR1 ligand-mediated axonal growth inhibition and Nogo-induced apoptosis. LOTUS upregulated the expression of neurotrophic factors including BDNF, NGF, and NT-3 (57). *In vivo*, LOTUS-NS/PCs extended neuronal fibers successfully into rostral and caudal host spinal segments, indicating enhanced axonal elongation, reduced apoptosis, increased neurotrophic secretion, and improved locomotor recovery—providing significant benefits for cell-based SCI repair (58).

- Wnt4

Inhibition of JMJD3-mediated SAPK/JNK inactivation is required for endogenous NPC differentiation toward the oligodendrocyte lineage, representing a potential epigenetic strategy to induce endogenous repair in mature mammals. Li et al. demonstrated that Wnt4 promotes neuronal differentiation of NSCs by activating β-catenin and MAPK/JNK signaling and suppressing Notch pathway activation through downregulation of NICD expression—a key factor that normally inhibits neuronal differentiation. *In vivo* transplantation of Wnt4-modified NSCs effectively promoted spinal cord repair, enhanced neuroregeneration, and significantly improved hindlimb motor function recovery (59).

### 4.3 NPCs overexpressing pro-survival factors

Genetic modification of NPCs with pro-survival molecules can enhance graft survival by improving cellular viability and modulating the injury microenvironment, thereby contributing to improved functional recovery after SCI (60).

- Galectin-1

In an *in vivo* adult common marmoset model of cervical contusive SCI, Gal-NS/PCs were transplanted intraspinally at 9 days post-injury. Compared with unmodified NS/PCs, Gal-NS/PCs achieved better behavioral recovery in spontaneous movement, grip strength, and treadmill performance. Histologically, grafted cells survived and differentiated into neurons, astrocytes, and oligodendrocytes, with significant improvements in myelinated area, corticospinal fibers, and serotonergic fibers. These findings support Gal-1 overexpression as a strategy to enhance the therapeutic efficacy of NS/PC transplantation in primate SCI (61, 62).

- Engineered Tropomyosin Receptor Kinase C (TrkC)

In an *in vivo* rat spinal cord transplantation study, TrkC-engineered neural precursor cells showed markedly improved survival when combined with NT-3 stimulation, reaching nearly 100% survival vs. 30–50% in the absence of TrkC or NT-3. NT-3 exposure, delivered either by *in vitro* pretreatment or *in vivo* application via gelfoam or Oxycel, also enhanced migration of transplanted cells within the spinal gray matter and reduced astrocytic differentiation. In addition, decreased cavitation and increased β-tubulin-positive fibers were observed near the graft, suggesting improved tissue preservation and axonal growth support (63, 64).

## 5 Combination strategies of NPCs with biomaterials

### 5.1 Types of combined biomaterials and their major mechanisms of action

The combination of NSPCs/NSCs with biomaterials has become an important strategy for spinal cord injury (SCI) repair. Current material platforms mainly include scaffold-based materials, functionalized hydrogels or nanomaterials, drug delivery systems, and emerging bioengineering approaches such as exosomes, gene editing, and optogenetics. Overall, scaffold-based materials mainly provide three-dimensional structural support and tissue bridging; functionalized hydrogels and nanomaterials emphasize local sustained release and microenvironmental modulation; drug delivery systems influence the fate of NSPCs through specific signaling pathways; and emerging engineering strategies further enhance regenerative regulation and the precision of therapeutic intervention.

### 5.2 Classification by mechanism of action

Mechanistically, biomaterial-assisted NSPC therapies exert their effects mainly by promoting neural regeneration and axonal extension, regulating the inflammatory and immune microenvironment, inhibiting glial scar formation, and inducing directed differentiation. Scaffolds and hydrogels can provide an adhesive and guidance-supportive environment, while combined growth factors promote axonal regeneration and remyelination. Some functionalized materials can attenuate inflammatory responses and improve the transplantation microenvironment. Anti-scarring strategies can enhance cell migration and axonal penetration. In addition, small molecules or functionalized scaffolds can guide NSPCs toward repair-favorable cell lineages through the regulation of key signaling pathways.

### 5.3 Classification by application scenario

Different combinatorial strategies may offer distinct advantages at different stages of injury. In acute or subacute SCI, anti-inflammatory protection and early optimization of the injury microenvironment are of primary importance; therefore, anti-inflammatory nanosystems or factor-releasing hydrogels may be more suitable. In chronic SCI, tissue bridging, axonal regeneration, and neural circuit reconstruction become the main therapeutic goals, making scaffold-based strategies combined with NSPCs and growth factors more promising. As research advances, some of these combinatorial approaches have gradually entered the preclinical or early translational stage, with the focus shifting from short-term reparative effects to long-term safety and host integration.

### 5.4 Advantages and challenges of combinatorial strategies

Overall, biomaterial-assisted NSPC therapies are superior to monotherapies in improving cell survival, optimizing the microenvironment, and promoting neural regeneration and functional recovery, thereby demonstrating clear synergistic advantages. However, this field still faces several challenges, including biomaterial biocompatibility, the matching of degradation rates, safety, and long-term integration. In addition, the translation of encouraging animal data into clinical practice remains limited by the substantial heterogeneity of human SCI. Future studies should further optimize material design, define the optimal timing of intervention, and strengthen long-term evaluations of efficacy and safety (Table 4).

**Table 4:** Classification, mechanisms, and application scenarios of combinational NSPC-based strategies.

Category	Representative examples	Main mechanism	Typical application
Scaffold-based materials	Collagen, hydrogels, porous nanofibers	Provide 3D support and promote adhesion, migration, and differentiation	Bridging and reconstruction in chronic SCI
Functionalized hydrogels/nanomaterials	IGF-1 hydrogel, magnetic nanoparticles plus methylprednisolone, IL-10-releasing hydrogel	Sustain factor release and modulate inflammation/microenvironment	Early protection in acute/subacute SCI
Drug-delivery systems	Polymer-ROCK inhibitor, EGFR antibody-functionalized scaffold	Regulate key signaling pathways and guide differentiation	Regeneration and lineage control
Anti-scar strategies	ChABC combined with NSPC transplantation	Degrade CSPGs and facilitate migration and axonal penetration	Scar remodeling in chronic SCI
Emerging bioengineering strategies	Exosomes, gene editing, optogenetics	Enhance regenerative capacity and circuit modulation	Preclinical precision intervention
Translationally oriented strategies	Scaffolds plus NSPCs plus growth factors/controlled-release systems	Improve survival, integration, and functional recovery	Preclinical and early translation

## 6 Clinical trials

### 6.1 Overview of clinical trials

**Figure 4:**
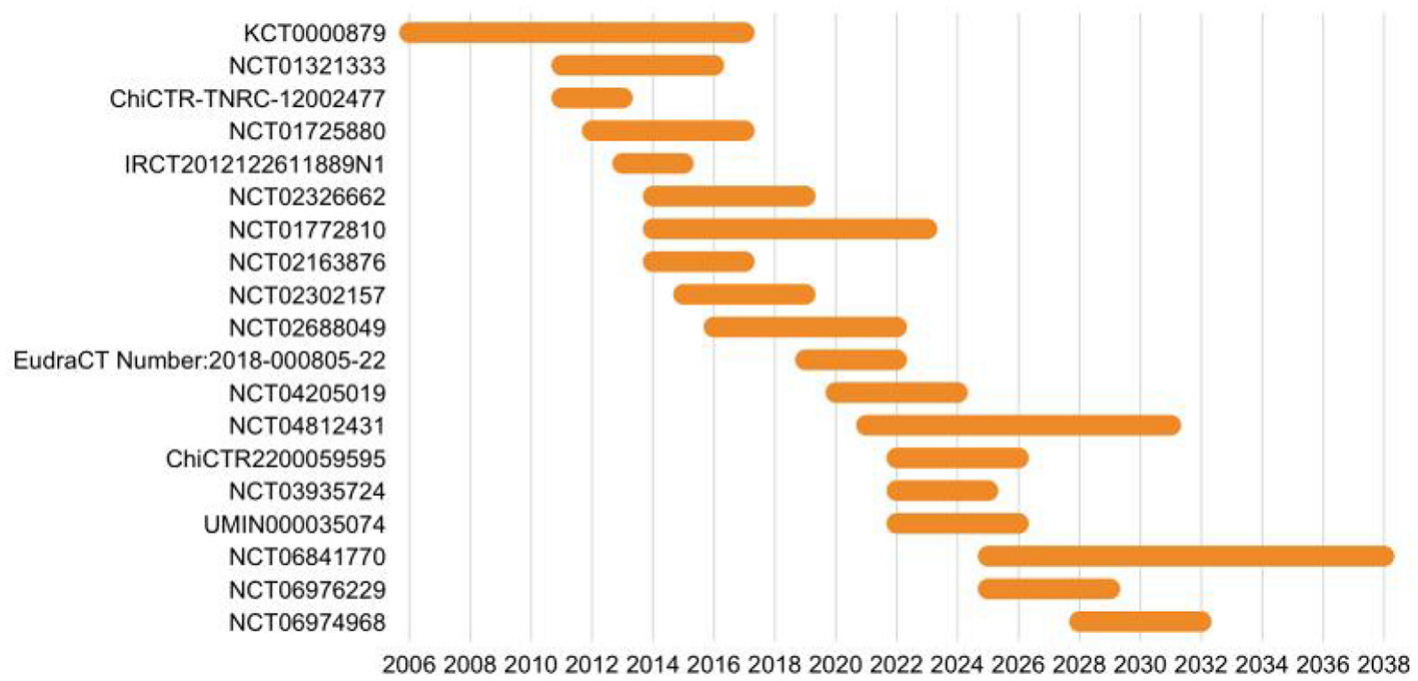
Start and finish time of each clinical trial involving NP/SCs.

**Figure 5:**
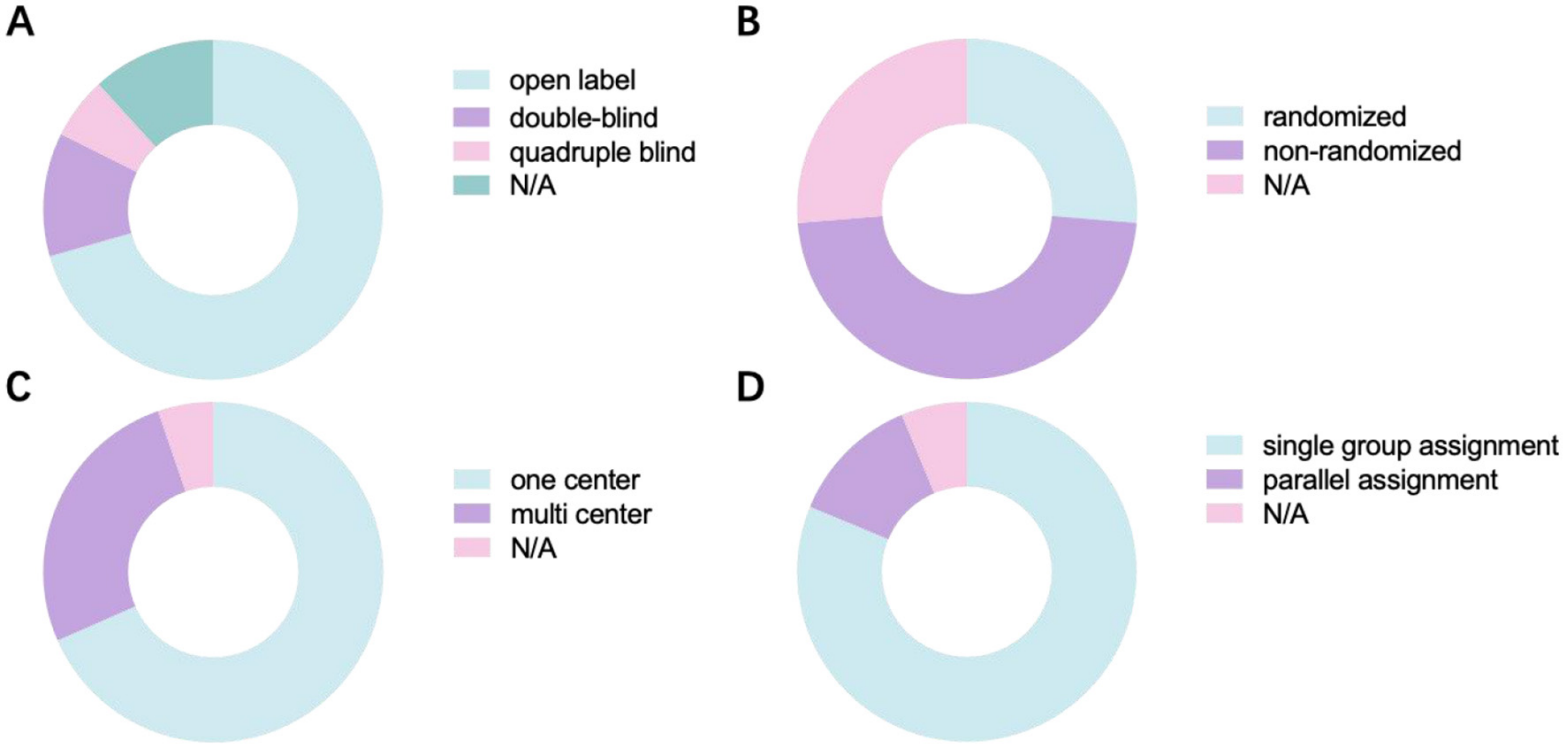
Study design of clinical trials involving NP/SCs for SCI. masking **(A)**, allocation **(B)**, number of centers **(C)**, intervention model **(D)** of clinical trials.

**Figure 6:**
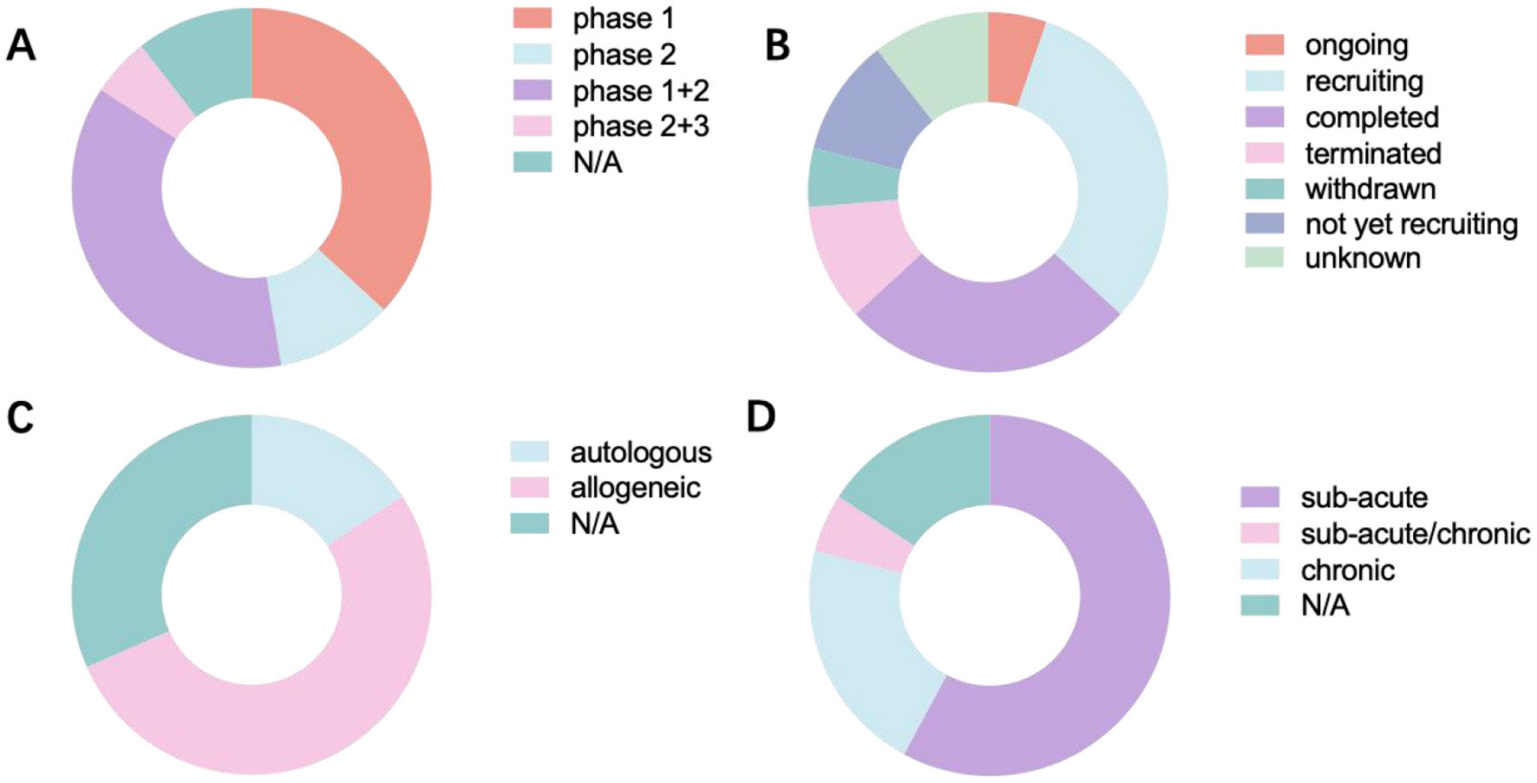
Characteristics of clinical trials involving NP/SCs for SCI. phase of clinical trials **(A)**, status **(B)**, origins of NP/SCs **(C)**, stage of SCI **(D)**.

A systematic search was conducted to identify clinical studies assessing NPC-based therapy for SCI (41). A total of 19 clinical trials were included (Table 3). The initiation and completion dates of these trials are shown in Figure 4. These studies were conducted across 10 countries, with China contributing the largest number of trials and a planned enrollment of 491 participants. Among the included studies, 12 trials were open-label and 2 were double-blind (Figure 5A). Five trials employed randomization, while 8 were non-randomized (Figure 5B). Thirteen studies were single-center and 6 were multi-center (Figure 5C). Additionally, 13 trials used single-arm or parallel-arm allocation (Figure 5D). Regarding trial phases, 6 studies (25%) were Phase I, 3 studies (13%) were Phase II, and 7 studies (44%) were Phase I/II combined. Only one trial progressed to Phase III, indicating that most studies remain in early development stages without late-phase validation (Figure 6). Of the 19 clinical trials, 3 used autologous NPCs, 10 used allogeneic NPCs (Figure 6C), and 6 lacked clear descriptions of cell sources. Consistent with the pathophysiological characteristics of SCI, all trials focused on patients in the subacute or chronic phase (Figure 6D and Table 5).

**Table 5:** Clinical trials using NP/SCs to treat SCI.

No.	Injury cite	Cell type	Cell origins	Delivery	Dosage	Inclusion criteria/ASIA	Outcome	Follow up/time frame	Country
1	SCI	With 3D matrix/neural stem cell transplantation	Autologous	Intraspinal and intrathecal injection	N/A	A/B	Feasibility and safety: side effects and surgery procedure	3, 6,12, 24 months and 3 years	Moscow/Russia
2	C5-T12	NeuroRegen scaffold/neural stem cells	N/A	Intraspinal and intrathecal injection	1 × 10^7^	A/B	Safety and tolerability	1, 3, 6, 12, 18 and 24 months	Tianjin/China
3 (65)	SCI T2-T12; C5-C7	Human spinal cord stem cells	N/A	Surgical implantation	1.2 × 10^6^	AIS-A complete	Safety and scientific validity	54 months	San Diego, California, United States
4	C4; C5-C7	Neural precursor cells derived from human embryonic stem cell line	Allogeneic	intrathecal injection (5 areas)	N/A	AIS-A	Safety and exploratory efficacy	Weeks 1, 2, 4, 8, 12, 24, 48, and 72 after surgery (1 year and 5 months)	Seoul, Korea
5	C5-C7	HuCNS-SC cells (human neural stem cells)	Allogeneic	Intramedullary transplantation	2 × 10^8^	Association impairment scale (AIS) grades B or C	Safety and efficacy	N/A	United States
					/4 × 10^8^				
6	T2-T11	HuCNS-SC	Allogeneic	Intramedullary transplantation	N/A	N/A	Safety and efficacy	4 years	Zurich, Switzerland
7	C5-T12	Neuro-cells	Autologous (one batch/one patient)	Intrathecal	N/A	Traumatic complete (AIS grade A) and incomplete (AIS grade B/C)	The safety	3 months, 12 months and 24 months	Spain
8	T2-T11	HuCNS-SC cells	Allogeneic	Intramedullary	N/A	AIS-A,B,C	Safety and preliminary efficacy	24 months	Calgary, Alberta, Canada; Toronto, Ontario, Canada; Zurich, Switzerland
9	T; C	Human neural stem cells	N/A	Transnasal injection	N/A	ASIA grade B-D	Safety and preliminary efficacy	N/A	Shanghai, China
10	N/A	Stem cells	N/A	Local transplantation	N/A	AIS-A	Efficacy	N/A	Beijing, China
11	C6-T12	Neural cells	N/A	Intrathecal	N/A	AIS-A,B,C	Safety and efficacy	Day 0, 90 and 180 of the study;	Spain
								Day 0, 180 and 365 of study.	
12	N/A	Human neural stem cells	Allogeneic	Injected into the lesion site	5 × 10^4^	AIS-A	The safety, tolerability and neurological status	6 and 12 months	Seoul, Korea
13	C4-T12	Neuro-cells	Autologous	Intrathecal injection	N/A	AIS-A,B,C	The safety and efficacy	1y	Spain
14	C4-C7	AST-OPC1 (oligodendrocyte progenitor cells)	Allogeneic	Intrathecal injection	2 × 10^6^	AIS-A	The safety of cross sequential escalating doses of AST-OPC1	1y (30, 60, 90, 180, and 365 days)	United States
15	T1-T10	Neural stem cell	N/A	Intraspinal cord upper and lower part of lesion	N/A	Definite paraplegic classification supported Franklin (A)	The safety and efficacy	6 months; 1y	Tehran
16	C3-T10	hiPSC-NS/PCs	Allogeneic	Intrathecal injection	2 × 10^6^	AIS-A	The safety and efficacy	52 weeks	Japan
17	C4-T10	LCTOPC1	Allogeneic	Injected into the lesion site	1 × 10^7^	AIS-A,B,C	Frequency and Severity of Adverse Events	1 month; 3 months	United States
18	C4-L2	XS228	Allogeneic	intrathecal injection	5 × 10^7^/1.5 × 10^8^	AIS-A,B,C	The incidence of adverse events (AEs) and serious adverse events (SAEs)	28d; 90d; 180d; 270d; 360d	China
19	C4-L2	XS228	Allogeneic	Intrathecal injection	N/A	N/A	The incidence of adverse events (AEs) and serious adverse events (SAEs)	28d; 90d; 180d; 270d; 360d	China

### 6.2 Clinical trial outcomes

To provide a direct comparison of the clinical performance of different neural stem cell products for SCI, this study summarized and compared the key clinical results of five major products: HuCNS-SC, LCTOPC1, NSI-566, hESC-OPC, and XS228.

#### 6.2.1 Study design

HuCNS-SC has been investigated in multicenter randomized Phase II and Phase I/II clinical trials (NCT02163876, NCT01321333). A total of 43 patients with chronic SCI were enrolled, including 12 thoracic (T2–T11) cases in NCT01321333 and 31 cervical (C5–C7) cases in NCT02163876, with the primary aim of evaluating safety and potential efficacy. LCTOPC1 (also known as GRNOPC1 or AST-OPC1) was assessed in a Phase I/IIa single-arm dose-escalation study involving 25 adults with subacute (21–42 days post-injury) cervical (C4–C7) AIS A SCI (NCT02302157), focusing on feasibility and safety. NSI-566 has undergone a single-center Phase I safety study (NCT01772810), in which 8 patients with chronic AIS A SCI were followed for 18–27 months to evaluate dose-escalation safety. The first hESC-OPC Phase I trial approved in 2010–2011 was terminated prematurely due to funding limitations, and therefore lacked systematic patient enrollment. A Japanese study (UMIN000035074) planned for subacute SCI administration has been postponed due to the COVID-19 pandemic. Notably, two recently launched trials in 2025 target subacute AIS A–C SCI. Compared with LCTOPC1 (NCT06841770), which uses single local administration mainly for AE and SAE monitoring, XS228 (NCT06976229) includes both single-dose and multiple-dose administration and has also pre-registered a Phase II trial (NCT06974968).

#### 6.2.2 Cell sources

HuCNS-SC: human fetal spinal cord–derived neural stem cells.

LCTOPC1: hESC-derived oligodendrocyte progenitor cells.

NSI-566: adult spinal cord–derived neural stem cells.

hESC-OPC: oligodendrocyte progenitor cells derived from hESCs.

XS228: motor neuron cells derived from iPSCs.

#### 6.2.3 Dosing regimens

HuCNS-SC was delivered by multiple intramedullary injections with doses of 2 × 10^8^ or 4 × 10^8^ cells to evaluate maximal tolerance.

LCTOPC1 was administered as a single intramedullary injection with three dose levels: 2 × 10^6^, 1 × 10^7^, and 2 × 10^7^ cells.

NSI-566 was injected at six sites surrounding the lesion, with 2 × 10^5^ cells per site (total 1.2 × 10^6^ cells).

hESC-OPC similarly utilized intramedullary administration.

XS228 used single intrathecal injection for the SAD cohorts (5 × 10^7^ or 1.5 × 10^8^ cells) and repeated intrathecal injections on Days 1, 15, 29, and 43 for the MAD cohorts with the same doses, following a 3 + 3 rule-based dose escalation.

#### 6.2.4 Safety evaluation

All Phase I/II studies demonstrated favorable tolerability.

For HuCNS-SC, all related adverse events (AEs) were mild to moderate in severity with no ≥Grade 3 cell-related serious AEs observed, and a 5-year long-term follow-up further confirmed its safety.

For LCTOPC1, among 25 participants, 534 AEs were reported: 32 associated with transplantation procedures or immunosuppression, 502 related to expected SCI complications; two Grade 3 events (CSF leakage and wound infection) fully resolved after treatment.

For NSI-566, no ≥Grade 3 transplantation-related AEs were documented.

For hESC-OPC, no severe complications directly attributable to the cells were reported.

For XS228, safety results have not yet been released.

#### 6.2.5 Functional outcomes

HuCNS-SC showed mild improvements in upper extremity motor scores (UEMS) and ISNCSCI scores, although results did not reach pre-specified efficacy endpoints.

LCTOPC1 demonstrated promising motor recovery: at 1-year follow-up, 96% (21/22) of subjects exhibited at least one AIS grade improvement, and 32% (7/22) improved by two grades or more, with notable gains in UEMS and GRASSP scores.

NSI-566 showed early sensory or motor function improvements in 3 out of 4 assessable patients during 18–27 months of follow-up, and MRI revealed graft-associated axonal sprouting.

For hESC-OPC, limited imaging data suggested possible axonal regeneration, but efficacy remained unclear due to early trial termination.

For XS228, efficacy data have not yet been reported.

- HuCNS-SC

HuCNS-SC is a proprietary human neural stem cell product being developed as a potential cell therapy for NCI (NCT00337636), AMD (NCT01632527), PMD (NCT01005004), and SCI. According to our search, Trials No. 5 and No. 8 investigated HuCNS-SC for SCI treatment. In 2012, StemCells Inc. conducted a Phase I clinical trial evaluating human fetal neural stem cell transplantation for chronic SCI. Twelve months of follow-up confirmed the safety and potential efficacy of HuCNS-SC transplantation (65). The No. 8 clinical trial further demonstrated the safety of HuCNS-SC transplantation into the chronically injured cervical spinal cord, with 12-month clinical follow-up providing additional support (29). However, all clinical trials involving HuCNS-SC were eventually terminated. Although the termination was not due to safety concerns, no conclusive clinical efficacy outcomes have been reported (30).

- LCTOPC1

LCTOPC1 (also referred to as GRNOPC1 or AST-OPC1) is derived from a single human embryonic stem cell (hESC) clone through a proprietary cell renewal process and is intended for single-dose administration to treat traumatic SCI. Data from a thoracic and cervical SCI clinical trial (Trial No. 14) demonstrated that hESC-derived therapies can be safely delivered into the spinal cord. Individual results from 30 enrolled participants provided evidence to regulatory bodies supporting the feasibility of applying an hESC-derived therapeutic intervention in patients with cervical injury levels up to C4 and AIS grade A or B. During 1-year follow-up for complications, all 25 treated participants experienced at least one AE, with a total of 534 AEs reported. Among them, 32 AEs were considered treatment-related and 502 were judged to be expected SCI-related complications. Twenty-nine serious AEs occurred, and two Grade 3 serious AEs (one cerebrospinal fluid leak and one bacterial infection) were resolved after appropriate medical intervention and were attributed to the injection procedure and tacrolimus-based immunosuppression. Based on the collected data, further assessment of LCTOPC1 efficacy in larger trials was considered warranted (66). In the treated cohort, 21 of 22 (96%) participants showed at least a one-grade improvement in neurological function on at least one side of the body, and 7 of 22 (32%) showed a two-grade or greater improvement after 1 year of follow-up (67).

In Cohort I, dose escalation established procedural safety, tolerability, and feasibility. However, although midterm analyses in Cohorts I and II showed trends toward improvement in UEMS and GRASSP motor scores, the magnitude of improvement did not meet the predefined efficacy threshold required to justify further development, ultimately leading to early trial discontinuation.

- NSI-566

NSI-566 represents another FDA-authorized human neural stem cell line for clinical investigation. Previous Phase I and II studies in patients with amyotrophic lateral sclerosis (ALS) demonstrated favorable safety. A Phase I clinical trial implanting NSI-566 into the injury sites of four individuals with chronic AIS A thoracic SCI reported follow-up periods of 18–27 months. The treatment demonstrated good safety, tolerability, and signs of neurological improvement, without any serious transplantation-related AEs (27, 28, 68). These findings support continued dose-escalation studies in SCI patients.

Additionally, the first FDA-approved human neural stem cell transplantation Phase I study for chronic SCI (2014–2022) further demonstrated the safety of NSI-566 transplantation, showing dense axonal sprouting throughout the graft region. Transplantation of human neural progenitor cells into the injured cervical spinal cord was found to be safe and well tolerated, with moderate neurological benefits observed within 1 year after transplantation.

- OPC

Geron initiated a Phase I clinical trial using hESC-derived oligodendrocyte progenitor cells (OPCs) for SCI treatment. The study was approved in 2009, began enrolling patients in October 2010, and was terminated in 2011 due to financial constraints. Thomas Scott later reported trial-related considerations and provided recommendations for IRBs and clinical researchers reviewing early-phase experimental medicine involving cutting-edge therapies (69). Another trial (UMIN000035074/jRCTa031190228) in Japan was postponed due to the COVID-19 pandemic and is planned to begin recruiting once conditions allow (38).

- XS228

XS228 is an iPSC-derived cell therapy product independently developed by Sironax Biotech. The Phase I clinical study (NCT06976229) was approved earlier this year and began patient enrollment in March 2025. In parallel, a randomized, double-blind, placebo-controlled Phase II trial (NCT06974968) has been registered. Approximately 60 patients with subacute SCI (2–12 weeks post-injury) are planned for enrollment. Participants in the treatment arm will receive four intrathecal injections of XS228 via lumbar puncture. The primary objective is to evaluate improvement in motor function at 6 months.

### 6.3 Summary of clinical trials on NPCs

To date, the FDA has not approved any therapies that can induce neurological recovery following spinal cord injury, especially for patients initially classified as motor complete (AIS A or B), who have more severe impairment. Current clinical trials have provided essential safety data, offering a foundation for future development of novel stem cell-based or combination therapies for SCI. Notably, expanded trials approved in 2025 by K Pharma in Japan and the ongoing registration Phase I clinical study in China by Sironax Biotech are expected to provide stronger evidence regarding therapeutic efficacy.

However, the significance of current clinical studies lies not only in their preliminary demonstration of the feasibility and safety of NPC/NSC-based therapies, but also in the barriers they reveal to further clinical translation. The HuCNS-SC program indicates that acceptable safety alone is insufficient to support continued development, particularly when functional improvement remains modest. Likewise, the experience with LCTOPC1/AST-OPC1 suggests that even when some degree of neurological improvement is observed, further advancement may still not be justified if the predefined efficacy threshold is not met. In addition, the early termination of the Geron hESC-OPC trial and the delay of the Japanese hiPSC-NS/PC trial also highlight the importance of commercial sustainability and operational feasibility in the development of SCI cell therapies. Conversely, partially encouraging outcomes have also been observed in some trials. For example, the LCTOPC1 study mainly enrolled patients with subacute cervical AIS A injury, resulting in a relatively concentrated study population, and its cell product consisted of hESC-derived oligodendrocyte progenitor cells with a clearly defined therapeutic target. Although NSI-566 was evaluated in a small cohort, it focused more specifically on patients with chronic AIS A injury and showed graft-associated axonal sprouting together with a certain degree of neurological improvement. These findings suggest that a more clearly defined cell lineage and better host integration potential may be important prerequisites for generating positive efficacy signals. By contrast, the HuCNS-SC studies included both thoracic and cervical SCI patients, and the heterogeneity in injury level and functional endpoints increased the complexity of result interpretation, which may also have reduced the clarity of the efficacy signal. In addition, optimized delivery strategies, adequate follow-up, and sensitive functional outcome measures may further improve the likelihood of detecting meaningful neurological benefit. Therefore, early positive findings should not be interpreted simply as evidence of the absolute superiority of a given product, but rather as the result of interactions among cell characteristics, trial design, patient stratification, and evaluation methodology.

Beyond this, safety assessment should not be limited to procedure-related complications and short-term tolerability. Particularly for gene-modified NPC products, greater attention should be paid to long-term risks, including insertional mutagenesis, uncontrolled transgene expression, off-target biological effects, vector-related immunogenicity, clonal selection, and tumorigenic transformation. Overall, the conclusions of NPC-based clinical trials indicate that several key challenges remain, including difficulties in isolating purified differentiated cell populations, limited efficiency of *in vivo* cell-tracking systems, and only moderate survival rates of transplanted cells (70). Although encouraging outcomes have been observed in clinical studies, substantial challenges persist in advancing NPC therapies for SCI. Reliable and innovative strategies are urgently required to enhance their functional potential and therapeutic effectiveness.

## 7 Organoid transplantation

From the perspective of cell origin, spinal cord organoids are themselves derived from neural progenitor cell lineage cells generated through neural induction of embryonic stem cells or induced pluripotent stem cells. Under three-dimensional culture conditions, growth factor modulation, and engineered support systems, these NPC-lineage cells undergo further self-organization, regional patterning, and network formation, ultimately giving rise to organoids with spinal cord-like architecture and cellular heterogeneity (71, 72). Therefore, organoids are not an independent class of graft material separate from the NPC system, but can instead be regarded as an advanced three-dimensional form of NPCs.

Recent preclinical studies further support the therapeutic potential of organoid-based strategies in SCI. In rodent models, GelMA-human spinal cord organoid composites improved locomotor recovery, reduced lesion volume, enhanced neural fiber ingrowth, and attenuated glial scar formation and inflammation (73). Similarly, organoid grafts have shown better neural fiber integration and functional recovery than cell-free or NPC-loaded hydrogel scaffolds, suggesting that mature microtissue-like constructs may outperform simple dissociated cell delivery (74). In addition, 3D-printed organoid scaffolds can guide axonal growth and support relay circuit formation with host tissue, leading to significant motor improvement after transplantation. These findings indicate that organoid-based grafts may enhance neuronal survival, structural integration, and functional recovery in SCI (75).

Conventional single-cell NPC transplantation has already shown important therapeutic potential in spinal cord injury (SCI). However, transplantation in the form of dissociated single cells still faces several key limitations. In contrast, the major value of organoids lies in their ability to establish a relatively complex cellular composition before transplantation, which may improve tissue compatibility and functional integration between the graft and the host (76). Taken together, organoids should be viewed as an important complement to traditional single-cell NPC transplantation. At the same time, organoids can serve as an important platform for NPC research. Human spinal cord organoids generated from NPC lineages can provide a model that more closely resembles the human pathological context, thereby allowing evaluation of NPC induction efficiency and differentiation direction (75, 77). In addition, organoids may function as an advanced tissue-engineering carrier for NPC transplantation, shifting the therapeutic strategy from transplantation of dissociated NPCs alone toward transplantation of organoid-like grafts composed of NPCs. This transition may not only enhance the stability and controllability of cell transplantation, but also create new possibilities for more precise spinal cord tissue replacement in the future. Overall, the introduction of organoid technology is driving the application of NPCs in SCI repair from conventional single-cell replacement toward a new stage characterized by tissue organization, regional specification, and bioengineering. Future studies should further focus on optimizing NPC sources, constructing segment-specific spinal cord organoids, improving host-graft integration after transplantation, and establishing long-term safety evaluation, so as to promote the continued translation of NPC-related therapeutic strategies from basic research to clinical application (Table 6).

**Table 6:** Major methods for spinal cord organoid construction.

Construction method	How it is constructed	Advantages	Limitations
Stem cell induction and differentiation (77, 114, 115)	iPSCs, ESCs, or reprogrammed somatic cells are induced into NPCs and then regionally differentiated	It recapitulates spinal cord development and generates specific cell types	Maturation and stability remain limited
Scaffold- or hydrogel-based 3D culture (76, 116)	NPCs or spheroids are embedded in Matrigel, GelMA, or collagen scaffolds	It provides a 3D niche that supports survival and growth	Niche simulation is incomplete, and material safety needs optimization
3D bioprinting/modular assembly (76, 117, 118)	Complex tissue-like structures are built by bioprinting or module assembly	It offers strong spatial control and structural heterogeneity	The process is complex and may reduce cell viability

## 8 Conclusion

Due to the pathological characteristics of SCI, current clinical trials are primarily focused on the subacute and chronic phases (78). Although complete functional recovery remains out of reach with existing therapeutic approaches, NPC therapy has demonstrated the ability to reduce secondary deterioration of spinal cord function and modestly promote axonal regeneration, highlighting its therapeutic potential (79). However, optimal transplantation timing, maximum dosage, and dosing frequency remain controversial due to the limited availability of clinical data, necessitating further investigation (Table 4).

A major requirement for therapeutic advancement is meeting the demands of clinical translation. The transition from preclinical studies to human application remains challenging. Commonly used preclinical animal models of SCI do not fully mimic the complex pathological conditions of SCI patients, which vary significantly depending on injury characteristics such as spinal level, extent of tissue loss, and presence of mixed lesion types (80–83). An appropriate, consistent, and reproducible SCI model is therefore essential for better understanding the mechanisms of traumatic spinal cord injury and for evaluating the efficacy of experimental therapeutic interventions. As organoid technology continues to advance, its integration with SCI research holds substantial promise.

Furthermore, although NPC transplantation has shown favorable safety, potential risks such as excessive proliferation or tumor formation and graft-induced pain must be minimized before clinical translation. Standardized and reproducible protocols are needed to ensure sufficient cellular manufacturing under defined conditions. Minimum potency requirements, stringent cell characterization, and complete *in vivo* dose—response assessments of efficacy and toxicity must be established (84). Additional preclinical studies are required to further validate and enhance the safety and effectiveness of NPC-based therapies (85).

With the encouraging progress achieved in SCI basic research, the need to conduct effective clinical trials is increasingly urgent. This review has comprehensively evaluated current clinical studies and provided detailed analyses. Future trials should aim to improve therapeutic efficacy while ensuring safety. In addition, this review highlights advances in genetically enhanced cell therapies, underscoring the need for improved experimental design and refinement of transplantation procedures.

**Figure 7:**
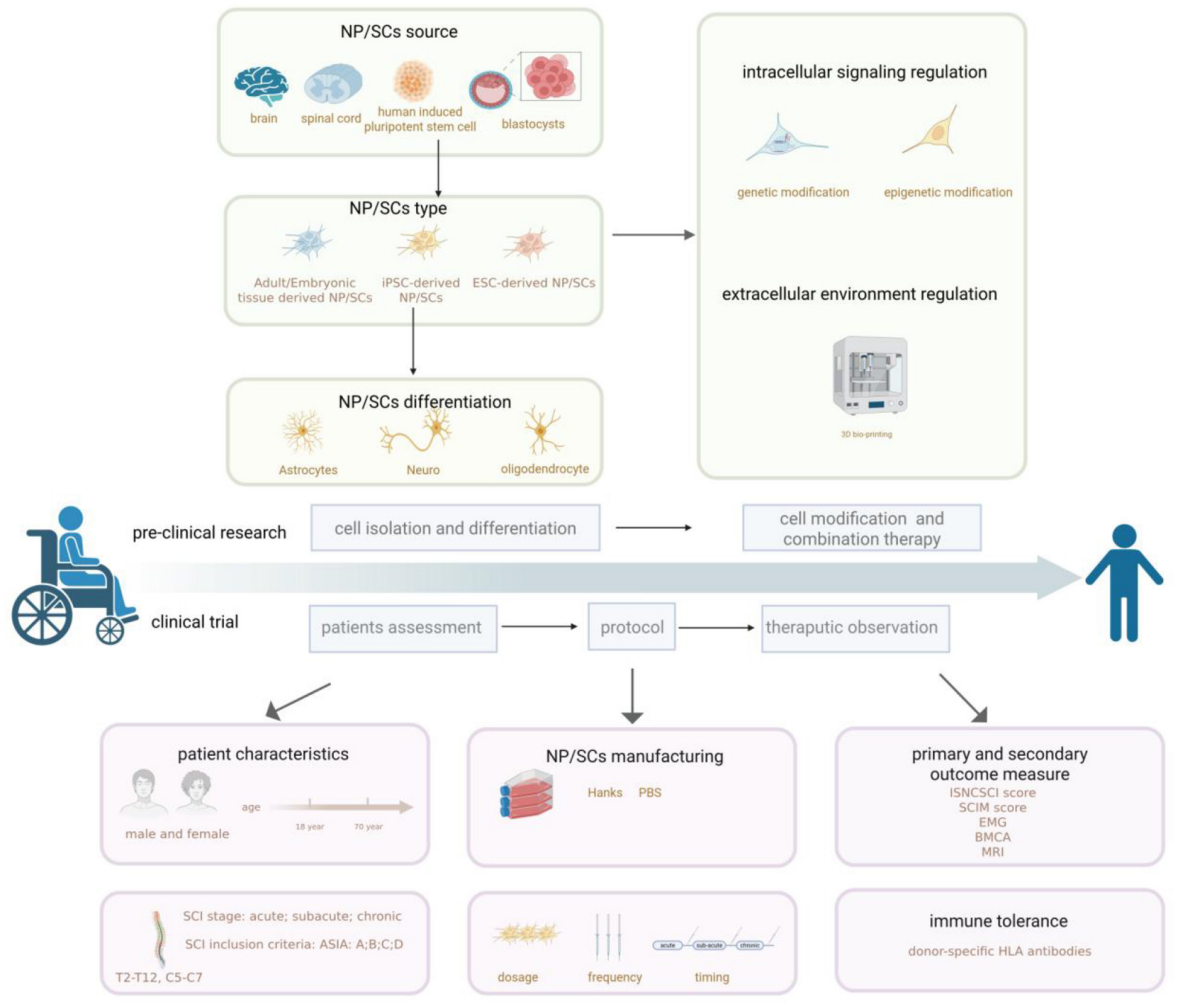
Analysis of preclinical research and clinical trials of NP/SCs in SCI.

In summary, this review provides a systematic overview of the current landscape of NPC-based treatments for spinal cord injury and identifies key areas requiring future improvement. Despite the challenges ahead, NPC therapy remains a highly promising strategy for achieving meaningful—and potentially complete recovery in patients with SCI (Figure 7 and Table 7).

**Table 7:** Characteristics of SCI patients who are more suitable for NP/SCs therapy.

Index	Remarks
Age	18–75y
SCI stage	Sub-acute; chronic
Injury level	AIS-A, B
Dosage	5 × 10^4^-4 × 10^8^/site—multi-sites
Times of cell administered	Multi-injection
